# IRVING S. WRIGHT AND VINCENT CRISTOFALO AWARD LECTURE

**DOI:** 10.1093/geroni/igz038.2046

**Published:** 2019-11-08

**Authors:** Stephanie Lederman, Hattie Herman

**Affiliations:** American Federation for Aging Research, New York, New York, United States

## Abstract

The Vincent Cristofalo Rising Star Award in Aging Research lecture will feature an address by the 2018 recipient, Nathan K. LeBrasseur, PT, PhD, of the Robert and Arlene Kogod Center on Aging, titled “Biomarkers of Senescent Cell Burden.” The Irving S. Wright Award of Distinction Lecture will feature an address by the 2018 recipient Pinchas Cohen, MD, of the USC Leonard Davis School of Gerontology, titled “Mitochondrial System Biology as a Window Into Diseases of Aging.” These awards are given by the American Federation for Aging Research, Inc.

